# Arterialised blood gas sampling in the critically ill: Correct tools for the job?

**DOI:** 10.4103/0972-5229.43675

**Published:** 2008

**Authors:** Suveer Singh

**Affiliations:** **From:** Intensive Care Unit, Magill Department of Anaesthesia, Intensive Care and Pain Management, Chelsea and Westminster Hospital NHS Foundation Trust, and Imperial College, London, UK

Sampling from blood has been performed for over 200 years. However clinically useful arterial blood gas sampling became a reality after the development of robust methods for measuring oxygen tension from humans in the 1940s. Thus the development of the Clark electrode and subsequent safe tools for sampling plasma, heralded an era of blood sampling, and analysis from which we have not looked back.[1] As with many new medical technologies of their time, it was borne out of a combination of the need for investigational improvements, inventive spirit and serendipity. Arterial Blood gas sampling has been the standard of care for monitoring acid-base disturbance for decades - a testimony to its value in diverse settings. Despite this, concerns remain regarding its risks to the patient from repeated same site sampling.[2] This in part led to the alternative approach of arterialized blood samples (AzB), using either an earlobe or finger pulp skin prick.[3] In theory AzB are more easily obtained than ABG, repeatable with better patient tolerance, and accurate. Thus it was surprising to know of the relatively poor uptake of this technique in the ambulatory setting.[4] Despite better utilisation in the UK, ongoing concerns regarding equipment, reliability and accuracy hamper its wider use.[5]

Quality control remains a crucial issue. Thus, a rigorous sampling technique, with adequate blood flow after topical vascularisation of the earlobe, sufficient volume and speed of collection, the appropriate sampling blade and rapid time to analysis has been proposed.[6]

What is known of its value in the clinical setting? The partial pressure of oxygen (*PaO_2_*) in AzB approaches but underestimates ABG due to venous admixture.[7] In the ambulatory setting, there appears to be a good correlation, both at rest, and after exercise in normal volunteers.[8] Values obtained for *pH, PCO_2_,* and *HCO_3_* show a tighter correlation to, and accuracy with ABG. As venous admixture is partly determined by the arterio-venous (A-V) difference in *PO_2_*, the greater this difference, the less accurate AzB values become, particularly for *PaO_2_*.[4,9]

The technique has been studied in the pediatric intensive care setting with favorable accuracy,[10] although it has not been universally adopted.

The study by Honarmand and Safavi in this edition of the IJCCM,[11] offers a relook at this long established but underused alternative blood gas sampling technique, in the setting of adult critically ill patients. They compared simultaneously collected AzB and ABG samples from 67 mechanically ventilated patients admitted with acute respiratory failure to a single centre general ICU, and looked at their correlation and accuracy with the 'gold standard ABG data. Using Bland and Altman regression analysis,[12] the mean difference between the two methods approached zero for *PaCO_2_* with a significantly good correlation (r=0.956), narrow limits of agreement and a range of ∼3kPa. This was also true for *pH*, and *HCO_3_*. However the limits were notably less for PaO*_2_* with a range of difference upto ∼6Kpa. Patients found sampling acceptable and there were no complications from this.

A significant proportion of these patients had head injury, without systemic illness. All were apparently normotensive although inotropic/vasopressor requirements are unclear. Thus the impact of underlying lung disease, deadspace ventilation and shunt fraction are unknown. These would all increase venous admixture and likely reduce the accuracy of AzB estimates of *PaO_2_*.

So does this study shed light on the implications for AzB as a clinically useful tool? A number of points emerge. First, it is an easily learnt technique that is acceptable to patients,[11] although not necessarily less uncomfortable than ABG sampling.[13] Second, it may provide clinically acceptable accuracy for the measurement of *pH, pCO_2_,* and *HCO_3_*, and trend analysis therein. However this can be offered equally well by standard venous blood sampling.[14] Third, it underestimates *PaO_2_* and its accuracy declines at higher Fractional inspired Oxygen concentrations (*FiO_2_*). So hypothetically, it would more accurately define PaO*_2_* in the most hypoxaemic patients, but this is the very group in who continuous inline monitoring of ABG is crucial to management.

It should be clear then, that an adult ICU setting is not the environment for the technique of AzB to be clinically useful. So is that the end of the road then for AzB? Perhaps not. Careful selection of the patient group in whom chronic hypoxaemia is established, and indwelling or multiple ABG sampling is undesirable, may lend itself to AzB for point-of-care testing. An acute-on-chronic deterioration may allow serial AzB trend analysis and facilitate management, perhaps in the acute medical setting. Another place for point-of-care AzB testing may be altitude, where the inaccuracies of venous admixture would be conveniently reduced due to relative hypoxaemia.

The value of socially acceptable, user friendly, rapid diagnostic testing tools in medicine that offer accurate clinical utility, continue to fuel research and development in the field of blood sampling. Whether a 40-year-old technique can be resurrected in a modern era will be down to further studies to identify its niche. That area will not likely be the adult critically ill.

So for once, it is not a case of the operator blaming his tools. Rather the tool has reached the limit of acceptable accuracy because of the intrinsic compensatory mechanisms that govern acid-base and gas-exchange homeostasis in the critically ill adult.
